# Divergent decoupling pathways for economic growth and NO_2_ emissions in China revealed by satellite observations

**DOI:** 10.1016/j.isci.2026.115352

**Published:** 2026-03-12

**Authors:** Jianbin Gu, Ziwei Chai, Ruimin Deng, Ying Zhang, Mingge Li, Liangfu Chen

**Affiliations:** 1 State Key Laboratory of Remote Sensing and Digital Earth, Aerospace Information Research Institute, Chinese Academy of Sciences, Beijing 100101, China; 2 Guangdong Ecological and Environmental Monitoring Center, Guangzhou 510308, China

**Keywords:** earth sciences, atmospheric chemistry, atmospheric observation, environmental science, remote sensing

## Abstract

China’s “dual carbon” goals require effective strategies to decouple growth from nitrogen dioxide (NO_2_) emissions, especially in transport and industry. Using TROPOspheric Monitoring Instrument (TROPOMI) satellite data (2019–2022) and high-frequency economic indicators, we employ a cascaded framework integrating Seasonal-Trend decomposition (STL), Granger causality, and cross-correlation functions (CCFs) to quantify sector-specific contributions. Our analysis reveals divergent pathways: (1) Passenger transport shows reduced short-term emission intensity (strong negative correlation, no Granger causality), aligning with rapid electrification under the 14th Five-Year Plan. (2) Freight transport maintains a strong positive linkage, highlighting a persistent diesel dependence bottleneck. (3) Industrial activity exhibits a moderating linkage, with 2022 NO_2_ concentrations stably below pre-pandemic levels despite full output recovery. These findings prioritize freight electrification, passenger-sector consolidation, and industrial transformation. The integrated framework provides a transferable paradigm for policy assessment in developing economies pursuing sustainable growth.

## Highlights

• Passenger transport achieved short-term NO_2_ decoupling via rapid electrification
• Freight transport exhibits zero-lag NO_2_ coupling stemming from diesel dependence
• Industrial NO_2_ stabilized below pre-COVID-19 peaks, showing emerging decarbonization
• Prioritize electrification and propel industrial CCUS to advance dual carbon goals

## Graphical abstract

**Figure:**
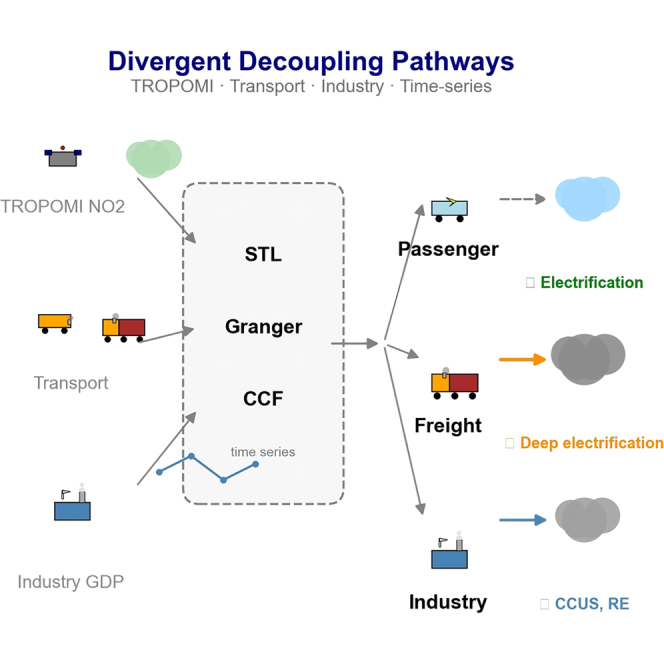

## Introduction

Nitrogen dioxide (NO_2_) poses severe public health risks across urban China, with extensive epidemiological evidence linking chronic exposure to respiratory and cardiovascular morbidity.^1^^,^^2^^,^^3^^,^^4^ Decades of rapid industrialization have entrenched NO_2_ emissions within economic growth paradigms—dominated by transportation networks and industrial production—creating persistent tensions between developmental imperatives and environmental protection.^5^^,^^6^^,^^7^ Resolving these tensions is now critical under China’s dual carbon goals (2030 peak; 2060 neutrality), demanding urgent strategies to decouple emissions from economic expansion.

Satellite remote sensing has revolutionized emission monitoring,^8^^,^^9^^,^^10^^,^^11^^,^^12^ with TROPOspheric Monitoring Instrument (TROPOMI)’s 3.5 × 5.5 km^2^ resolution enabling unprecedented tracking of pollution hotspots.^13^^,^^14^^,^^15^ Yet, fundamental knowledge gaps continue to impede policy optimization: sector-specific drivers (e.g., freight vs. passenger transport) remain poorly quantified, while time-lagged responses to interventions like electric vehicle (EV) adoption lack empirical validation. These limitations obstruct strategic resource allocation under China’s 14th Five-Year Plan (FYP, 2021–2025), which enacts rigorous measures including nationwide EV subsidies and industrial ultra-low emission standards.^16^

Three unresolved uncertainties hinder progress: quantifying passenger transport’s decoupling potential from NO_2_ emissions; mitigating stubborn freight-related pollution despite automation; and verifying industrial decarbonization rates against production scaling. Without addressing these gaps, China’s green transition risks inefficiency and delayed climate targets.

To bridge this divide, we integrate TROPOMI-derived NO_2_ data from 2019 to 2022 with high-frequency economic indicators in a cascaded analytical framework. This period encompasses unprecedented, non-cyclical shocks—most notably the COVID-19 pandemic and associated lockdowns—which induced extreme volatility in both economic activity and emissions. While the relationships observed during such a turbulent interval may differ from those under stable, long-term conditions, it provides a unique high-stress natural experiment to examine short-term system dynamics. Employing time-series decomposition, Granger causality, and cross-correlation functions (CCFs), we aim to diagnose the short-term associative dynamics and emission intensity signals between sectoral activity and pollution levels. This work seeks to establish responsive sectoral linkages, quantify policy-related temporal dynamics, and evaluate industrial decarbonization signals, thereby delivering actionable intelligence for China’s low-carbon transition while offering a transferable paradigm for developing economies pursuing sustainable growth.

## Results and discussion

### Spatiotemporal dynamics of NO_2_ concentrations (2019–2022)

**Figure 1:**
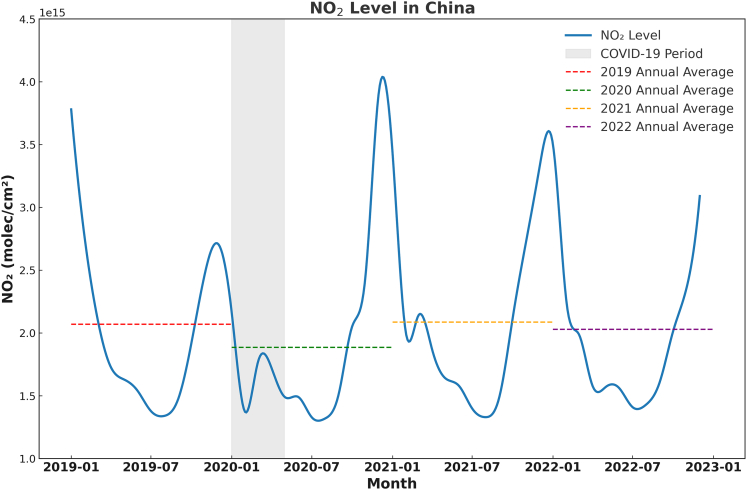
Monthly mean tropospheric NO_2_ VCDs (10^15^ molecules/cm^2^) over China (2019–2022) derived from TROPOMI observations The blue line depicts monthly averages; dashed colored lines indicate annual means. The shaded region marks the period of stringent COVID-19 lockdowns (early 2020). Data are presented as monthly mean values. VCDs, vertical column densities; TROPOMI, TROPOspheric Monitoring Instrument.

**Figure 2:**
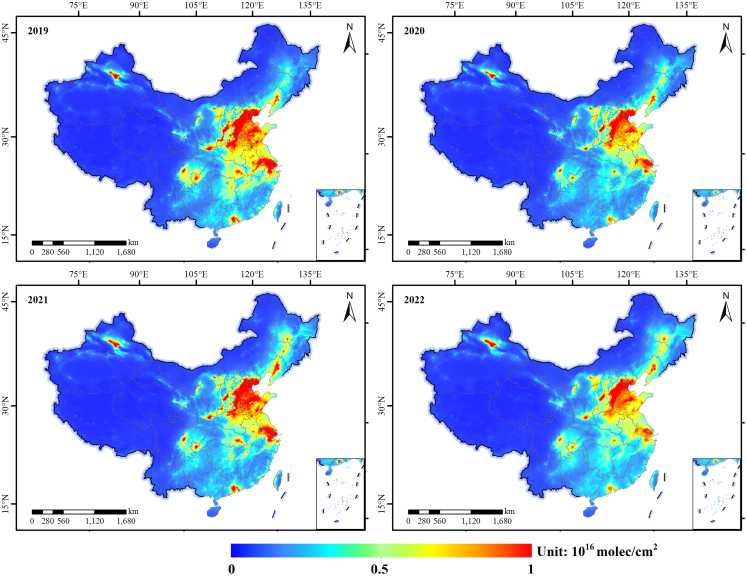
Spatial distribution of tropospheric NO_2_ VCDs (10^16^ molecules/cm^2^) across China (2019–2022) in January each year, based on TROPOMI data Persistent hotspots occur over major urban-industrial regions (NCP, YRD, and PRD). Each map shows the monthly mean value for the respective January. VCDs, vertical column densities; TROPOMI, TROPOspheric Monitoring Instrument; NCP, North China Plain; YRD, Yangtze River Delta; PRD, Pearl River Delta.

Satellite observations reveal distinct seasonal and spatial patterns in tropospheric NO_2_ vertical column densities (VCDs) over mainland China between January 2019 and December 2022 (Figures 1 and 2). A pronounced seasonal cycle dominates the temporal trend, characterized by peak concentrations during winter months (December–February) and minima in summer (June–August). This pattern is primarily driven by elevated emissions from coal-based heating systems under prevailing stagnant atmospheric conditions that limit pollutant dispersion during winter, contrasted by enhanced photochemical removal and stronger vertical mixing in summer.^17^

Spatially, NO_2_ pollution exhibits marked heterogeneity, persistently concentrating over major urban and industrial corridors. The highest burdens (>1.0 × 10^16^ molecules/cm^2^) consistently occur in the North China Plain (NCP), Yangtze River Delta (YRD), and Pearl River Delta (PRD) regions (Figure 2). These areas, hosting dense populations and intensive industrial activity, serve as primary hotspots for anthropogenic nitrogen oxide (NO_x_) emissions from transportation networks, power generation, and heavy manufacturing.

**Figure 3:**
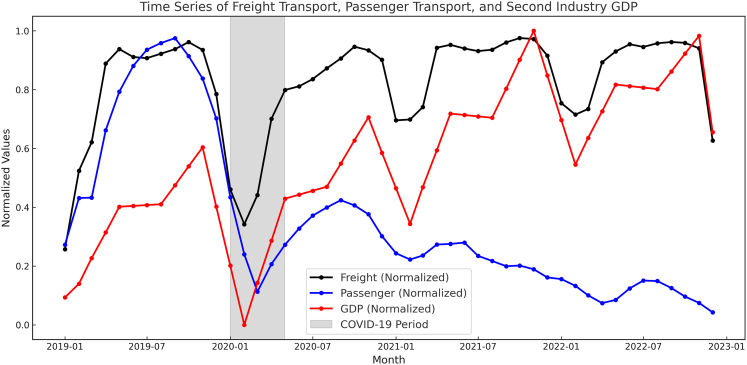
Normalized monthly time series for highway freight ton-kilometers, highway passenger-kilometers, and secondary industry value-added GDP in China (2019–2022) The shaded region denotes the COVID-19 lockdown period. Note the rapid rebound of freight and industrial output versus the sustained suppression of passenger activity during 2020–2021.

The COVID-19 pandemic induced a sharp, nationwide perturbation in early 2020.^13^^,^^18^ Stringent lockdown measures resulted in an abrupt 32% decline in mean NO_2_ VCDs compared to that in the same period in 2019, unequivocally demonstrating the dominance of anthropogenic sources (Figure 1). This reduction was most pronounced within the core industrial and urban zones of the NCP, YRD, and PRD, aligning temporally with documented collapses in industrial output and mobility. Following the relaxation of restrictions in mid-2020, NO_2_ concentrations rebounded rapidly, regaining pre-pandemic levels by late 2020. Notably, however, the annual mean NO_2_ concentration for 2022 remained measurably below the 2019 peak (Figure 1), even as key sectors like freight transport and industrial output had recovered to or exceeded pre-pandemic levels (Figure 3). We note that overall economic activity, particularly in the passenger transport and service (tertiary) sectors, may not have fully regained its pre-pandemic trajectory. Therefore, the lower 2022 national mean alone cannot definitively prove economy-wide decoupling. Instead, this aggregate observation prompts a more nuanced, sector-specific investigation into differential emission dynamics.

### Differential recovery of transport and industrial activities

The COVID-19 pandemic triggered pronounced disruptions across China’s key economic sectors, with distinct recovery trajectories evident in freight transport, passenger transport, and secondary industry GDP (Figure 3). Monthly data from the Ministry of Transport and National Bureau of Statistics reveal stark contrasts in vulnerability and resilience.

Freight transport activity exhibited robust resilience. Following a sharp but transient decline during the strict lockdown phase of early 2020, volumes rebounded rapidly. By mid-2020, freight ton-kilometers had largely recovered to pre-pandemic baselines, driven by resurgent industrial demand and supply chain restocking. This swift recovery underscores the sector’s fundamental role in sustaining economic operations. Concurrently, secondary industry GDP mirrored this pattern, experiencing a severe contraction in early 2020 but achieving a near-complete recovery to pre-pandemic levels by early 2021. The rapid rebound in industrial output highlights its systemic importance and adaptive capacity.

In stark contrast, passenger transport suffered a deeper and more protracted downturn. Activity plummeted dramatically during the initial lockdown period and remained substantially suppressed throughout much of 2020 and 2021. Persistent travel restrictions, reduced commuting, and subdued demand for public transit and intercity travel hindered recovery. The sector’s slower rebound reflects its heightened sensitivity to public health interventions and behavioral shifts. This divergence—rapid recovery in freight and industry versus sustained weakness in passenger mobility—establishes a critical foundation for analyzing their differential impacts on NO_2_ emissions in the subsequent sections.

### Predictive relationships and time-lagged associations with NO_2_ emissions

**Figure 4:**
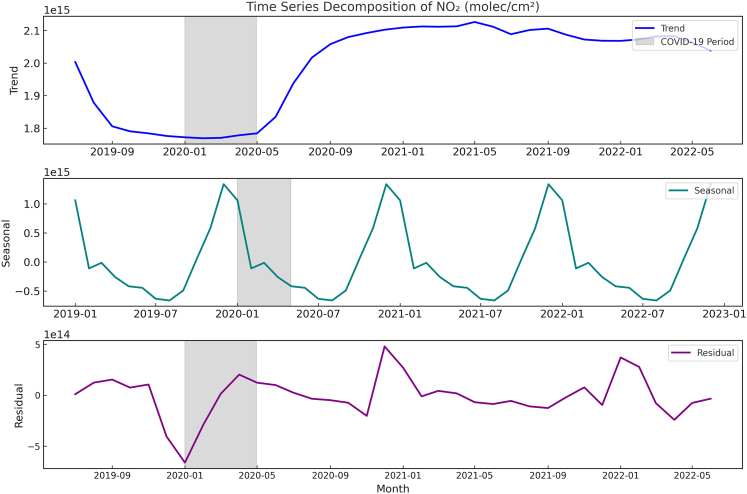
Decomposition of NO_2_ time series revealing anthropogenic trend signal (2019–2022) Components from STL: Observed data, long-term trend (reflecting economic/policy drivers), seasonal pattern, and residual variations. The trend component captures the sharp NO_2_ decline during COVID-19 lockdowns (shaded area) and subsequent recovery. STL, Seasonal-Trend decomposition using Loess.

We first isolated the anthropogenic drivers of NO_2_ variations by decomposing the time series using Seasonal-Trend decomposition using Loess (STL) (Figure 4). This method separated the signal into a long-term trend component (reflecting economic and policy influences), a predictable seasonal cycle (driven by winter heating and atmospheric stability), and short-term residual fluctuations. The trend component clearly captured the dominant perturbation: a sharp decline during early 2020 lockdowns followed by a robust recovery. This purified trend signal forms the critical basis for assessing causal links to economic activities.

Granger causality tests with Bayesian Information Criterion (BIC)-optimized lags were employed to examine the predictive relationships between the detrended economic activities and the NO_2_ trend component. It is important to note that Granger causality tests whether past values of one time series contain unique information for predicting current values of another; it indicates a predictive precedence or linkage within the observed data but does not establish structural or counterfactual causality. The results reveal a clear divergence: freight transport (*p* < 0.01) and secondary industry GDP (*p* < 0.01) significantly exhibit a Granger-causal relationship with variations in NO_2_ concentrations. In contrast, passenger transport exhibits no statistically significant predictive relationship with NO_2_ (*p* > 0.10) within this temporal framework. This absence of predictive power, despite the sector’s recovery trajectory, provides statistical evidence consistent with a reduction in the emission intensity of passenger mobility during the study period.

**Figure 5:**
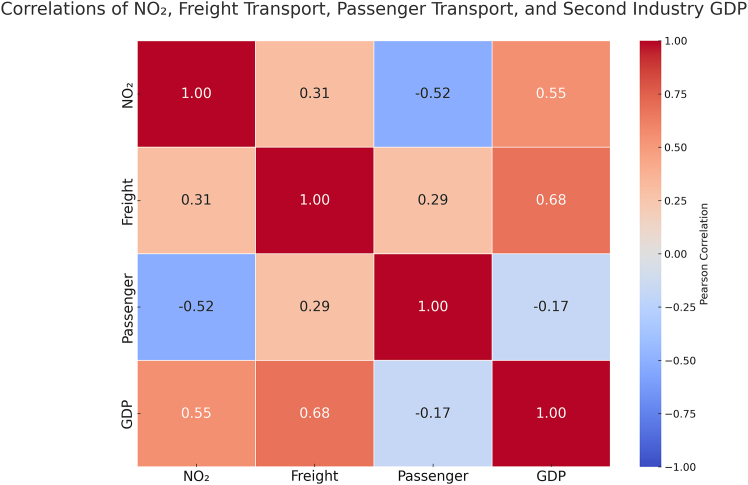
Divergent contemporaneous correlations between economic activities and NO_2_ pollution Pearson’s correlation coefficients between detrended NO_2_ concentrations and key economic indicators. Strong positive correlation with industrial GDP contrasts with the significant negative correlation for passenger transport, highlighting their divergent linkages to NO_2_ pollution. Data are presented as Pearson’s r values.

Pearson’s correlation analysis quantified the strength of contemporaneous relationships (Figure 5). Freight transport demonstrated a moderate positive correlation with NO_2_ levels (r = 0.31, *p* < 0.001). This finding aligns with the significant contribution of diesel-powered logistics to NO_2_ emissions. For example, according to the Ministry of Ecology and Environment (MEE) of China, diesel vehicles accounted for over 80% of total vehicular NO_x_ emissions across China in 2021.^19^ Secondary industry GDP exhibited a strong positive correlation (r = 0.55, *p* < 0.001), underscoring industrial production’s dominance. It should be noted that the monthly industrial GDP series is derived from quarterly data via interpolation and represents value-added, which may not perfectly align in time with the instantaneous emission processes; thus, this correlation primarily reflects a robust aggregate-level association, rather than a finely resolved temporal correspondence. Critically, passenger transport shows a pronounced negative correlation (r = −0.52, *p* < 0.001). This inverse relationship, combined with the absence of Granger causality, is consistent with a reduction in the sector’s emission intensity.

A critical consideration is whether the observed statistical pattern reflects technological mitigation or concurrent factors like pandemic-induced demand suppression. While the correlation strength and sign are suggestive, they cannot alone definitively isolate a reduction in emission intensity. The specific signature—a strong negative correlation where higher activity associated with lower pollution—is more consistent with decreasing emissions per unit of activity than with pure demand contraction. Furthermore, the non-significant Granger causality result is conditional on our model and does not rule out all associations. Crucially, the interpretation of reduced emission intensity gains strong, independent support from the unprecedented scale of transport electrification in China during the study period. EV sales surged from ∼1.2 million (2019) to ∼6.9 million (2022), and electrified railway mileage exceeded 114,400 km by 2022.^20^^,^^21^ This documented technological shift provides a concrete, mechanistic basis for a rapid decline in fleet emission intensity, making it the most plausible primary driver of the observed statistical disconnection. Therefore, evidence is mostly consistent with a technologically facilitated shift in the sector’s emission trajectory, while acknowledging the pandemic’s demand shock as part of the overall context.

**Figure 6:**
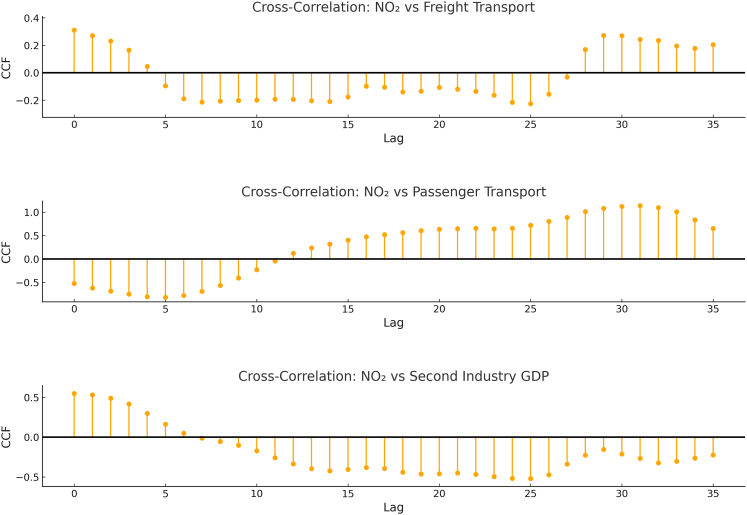
Distinct time-lagged impacts of economic activities on NO_2_ concentrations CCF analysis showing correlations at different monthly lags. Freight transport shows an immediate peak (lag 0), passenger transport reveals a short-term negative effect followed by a delayed positive peak (∼20 months), and industrial GDP exhibits sustained positive influence over multiple months. Lines represent the CCFs at each lag. CCF, cross-correlation function.

CCF analysis further mapped the temporal dynamics of these interactions (Figure 6). Freight transport exerts an immediate, strong positive impact on NO_2_, peaking at lag 0 month, though the effect decays rapidly. Passenger transport displays a significant negative correlation at short lags (0–6 months), reinforcing the evidence for reduced short-term emission intensity. It also reveals a delayed positive correlation at longer lags (∼15–25 months). This statistical pattern indicates that, within the observed post-pandemic recovery period, increases in passenger activity were associated with higher NO_2_ levels after a considerable delay. While this association does not imply causation or directly predict future trends—and must be interpreted with caution given the incomplete recovery of passenger mobility during the study timeframe—it highlights a distinct temporal dynamic. Secondary industry GDP demonstrates a persistent positive influence, with significant correlations spanning lags 0 to +12 months, highlighting the enduring challenge of industrial emissions mitigation.

Collectively, these analyses reveal fundamentally distinct temporal dynamics in the relationship between economic activities and NO_2_ concentrations. The results delineate a clear sectoral divergence: freight transport demonstrates an immediate association with NO_2_ levels, aligning with its persistent dependence on diesel combustion. Notably, this central role of freight transport in driving near-term NO_2_ variations is corroborated by an independent analysis of weekly cycles across China, which signified it as the primary factor explaining the lack of a significant weekend effect in ambient NO_2_.^22^ Passenger mobility exhibits a statistical pattern indicative of reduced short-term emission intensity—characterized by a lack of predictive linkage (Granger causality) and a strong negative correlation—which is consistent with the mitigating effect of rapid fleet electrification. Industrial output maintains a strong and prolonged predictive relationship with NO_2_ pollution, underscoring the structural challenge of industrial decarbonization. It is important to emphasize that these findings, derived from observational time-series analyses, identify robust predictive and associative patterns that highlight sectoral priorities. However, they cannot definitively isolate the causal effect of specific policy interventions from other concurrent macroeconomic or social factors. This converging evidence from different temporal perspectives pinpoints where policy attention and further causal investigation are most warranted.

### Policy effectiveness and pathways for sustained decoupling

Our findings provide strong empirical evidence aligned with the effectiveness of China’s targeted clean transportation policies under the 14th FYP. The case for passenger transport rests on a confluence of evidence: a distinct statistical signature of reduced short-term emission intensity, characterized by a strong negative correlation and absence of Granger causality, which is further corroborated by the unprecedented, coincident scale of fleet electrification during the study period. Crucially, this interpretation gains plausibility not from an assumption of full economic recovery but from divergent sectoral dynamics. The persistent positive linkage observed in freight transport under similar macroeconomic and restriction conditions suggests that the unique disconnection in passenger transport is not merely an artifact of broad demand suppression but points to a sector-specific transformation. Therefore, the observed pattern is mostly consistent with a technologically facilitated shift in emission trajectory, highlighting the tangible early returns of sustained investment in EVs and electrified rail under the current policy framework.

We explicitly acknowledge that our observational design cannot definitively separate the influence of this technological shift from concurrent behavioral changes induced by the pandemic, such as the popularization of remote work and travel restrictions, which undoubtedly suppressed mobility demand. While a scenario of pure demand suppression would be less consistent with the observed strong negative correlation—as the specific pattern where higher activity associates with lower pollution more readily aligns with a reduction in emission intensity per unit of activity—it remains impossible to fully disentangle and quantify their respective contributions. Therefore, although a causal attribution to policy-driven technology alone cannot be made from our aggregate analysis, the findings highlight passenger transport as a sector where the statistical signature of reduced emission intensity is clear.

Establishing a causal link would require research designs capable of constructing valid counterfactuals, such as quasi-experimental methods exploiting regional variations in policy implementation. Our integrated framework serves to identify such priority sectors and dynamic patterns, thereby providing critical targets for future causal evaluation. Furthermore, the delayed positive correlation was observed at longer lags (∼15–25 months) in the CCF analysis, while a statistical association that does not imply causation reveals a distinct temporal dynamic. Given the context of incomplete activity recovery, this pattern should be interpreted cautiously and does not directly predict future emissions. Nonetheless, it underscores that the recent gains are not automatically permanent. To prevent a potential renewal of upward pressure on NO_2_ from future mobility growth, consolidating and accelerating the ongoing technological transition remain the most direct and critical strategy.

In stark contrast, the immediate and strong positive impact of freight transport on NO_2_ levels highlights a persistent decarbonization bottleneck. The sector’s entrenched reliance on diesel-powered heavy-duty vehicles renders it a primary driver of near-term NO_2_ pollution, posing concurrent challenges for air quality and climate goals (given CO_2_ co-emissions). Current 14th FYP measures for freight greening are demonstrably insufficient. Achieving meaningful decoupling in the freight sector necessitates aggressive, sector-specific interventions: mandating accelerated electrification or hydrogen adoption for logistics fleets; enforcing stringent ultra-low emission standards (beyond China VI); implementing green corridor initiatives with priority charging/refueling; and introducing potent fiscal incentives for clean freight operators. Without such intensified efforts, freight emissions will impede both NO_2_ reduction and carbon peak objectives.

The strong, persistent linkage between secondary industry GDP and NO_2_ concentrations underscores the systemic challenge of industrial decarbonization. Yet, the observed stabilization of NO_2_ levels in 2022 below pre-pandemic peaks—despite full industrial output recovery—offers a promising signal. This suggests early-stage efficacy of 14th FYP industrial measures, including ultra-low emission retrofits, efficiency upgrades, and incremental renewable energy integration. Given NO_2_’s role as a tracer for fossil combustion, this stabilization may indicate nascent progress toward broader industrial carbon intensity reduction. Capitalizing on this momentum demands deeper structural shifts: scaling carbon capture, utilization, and storage (CCUS) for hard-to-abate sectors; accelerating renewable-powered electrification of industrial processes; and embedding circular economy principles to reduce primary production intensity. Continuous monitoring is vital to confirm and strengthen this potentially incipient decoupling trajectory.

Collectively, these results validate the strategic direction of China’s 14th FYP environmental policies while pinpointing critical gaps. The clear statistical signature of reduced emission intensity in passenger transport underscores the efficacy of targeted technological modernization under real-world conditions. Conversely, the freight sector’s inertia and industry’s persistent influence necessitate significantly bolder action. Our integrated analysis—leveraging high-frequency satellite data and economic indicators—provides a timely evaluation framework capable of tracking policy efficacy and informing iterative refinement. Importantly, our findings are derived from a period (2019–2022) marked by concurrent major shocks, most notably the COVID-19 pandemic. This context of extreme volatility provides a critical stress test of the economy-emission linkage, revealing how different sectors respond and recover under severe disruption. While the precise dynamics may differ in a stable, long-term growth environment, the insights into sectoral resilience and short-term adaptive capacity are vital for policy in an era facing increasing risks of non-cyclical shocks from health and climate. This work delivers actionable intelligence for optimizing China’s pathway toward its dual carbon goals. Prioritizing deep freight electrification, sustaining passenger sector innovation, and accelerating industrial process transformation are paramount. The methodology establishes a transferable paradigm for evidence-based air quality and decarbonization policy assessment in rapidly developing economies worldwide.

### Limitations of the study

This study provides a national-scale, integrated assessment of sectoral linkages to NO_2_ emissions, but several limitations should be considered when interpreting the findings. First, Granger causality and correlation analyses reveal predictive and associative patterns but cannot establish causality. Crucially, our aggregate design cannot separate the concurrent influence of technological modernization and pandemic-induced behavioral shifts (e.g., remote work) on passenger transport emissions—both remain plausible drivers of the observed reduction in emission intensity. This limitation highlights the need for future quasi-experimental designs to isolate causal mechanisms. Second, while monthly aggregation and seasonal decomposition mitigate the influence of recurrent meteorological cycles, they do not fully control for short-term synoptic anomalies, such as abrupt changes in wind speed or boundary layer height. These unresolved high-frequency meteorological variations may introduce residual noise into the derived associations between economic activity and NO_2_ concentrations. Third, our core sample period (2019–2022) encompasses both the acute disruption of the COVID-19 pandemic and a subsequent phase of recovery and adaptation. This temporal coverage allows the analysis to capture sectoral dynamics across a full cycle of economic shock and rebound, providing insights into short-term resilience. Nevertheless, the inclusion of this non-cyclical shock implies that the precise quantitative relationships identified here may differ from those that characterize a prolonged period of stable economic growth. Extending and validating these associations under more typical, long-term conditions will be an important objective for future research as longer time-series data become available.

## Resource availability

### Lead contact

Requests for further information and resources should be directed to and will be fulfilled by the lead contact, Ziwei Chai (chinaziweich@163.com).

### Materials availability

This study did not generate new unique reagents.

### Data and code availability

• Data

All datasets used in this study are publicly accessible and were available at the time of submission. Tropospheric NO_2_ VCD data from TROPOMI (January 2019–December 2022) used in this study are publicly available from the NASA Goddard Earth Sciences Data and Information Services Center (GES DISC) under the dataset “Sentinel-5P TROPOMI Tropospheric NO_2_ Column L2” (https://disc.gsfc.nasa.gov/datasets; https://doi.org/10.5270/S5P-9bnp8q8). China’s monthly freight ton-kilometers and passenger-kilometers were obtained from the Ministry of Transport open data portal: https://www.mot.gov.cn/shuju. Secondary industry GDP data are from the National Bureau of Statistics of China: https://data.stats.gov.cn. All datasets are also listed in the key resources table with their respective identifiers.

• Code

All original code used for STL, Granger causality analysis, and CCFs is provided as supplemental information (Document S1). The code enables full reproduction of the statistical analyses presented in this paper.

• Other items

Any additional information required to reanalyze the data reported in this paper is available from the lead contact upon request.

## Acknowledgments

We gratefully acknowledge the European Space Agency (ESA), the Royal Netherlands Meteorological Institute, and NASA for providing open-access TROPOMI and VIIRS data products essential for atmospheric monitoring. Our thanks extend to China’s Ministry of Transport and National Bureau of Statistics for making national-scale transportation metrics and industrial GDP data publicly available through their official portals. This research has been supported by the 10.13039/501100012166National Key Research and Development Program of China (grant no. 2023YFC3705801) and the Services of Guangdong Ecological and Environmental Monitoring Centre (ZXCG-2024-161).

## Author contributions

Conceptualization, J.G.; methodology, J.G.; data curation, R.D.; writing – original draft, J.G.; writing – review & editing, Z.C. and L.C.; validation, Y.Z. and M.L.

## Declaration of interests

The authors declare no competing interests.

## STAR★Methods

### Key resources table

**Table:** 

REAGENT or RESOURCE	SOURCE	IDENTIFIER
**Deposited data**		

TROPOMI NO_2_ Level 2 data	NASA GES DISC	https://disc.gsfc.nasa.gov/datasets; https://doi.org/10.5270/S5P-9bnp8q8
China transportation statistics	Ministry of Transport of China	https://www.mot.gov.cn/shuju
Secondary industry GDP	National Bureau of Statistics of China	https://data.stats.gov.cn

**Software and algorithms**		

Python version 3.13	Python Software Foundation	https://www.python.org
Python scripts for STL, Granger causality, CCF	This paper	Document S1 (supplemental information)

### Experimental model and study participant details

This study did not involve any experimental models or study participants. All analyses were conducted using publicly available satellite remote sensing data (TROPOMI NO_2_ observations) and aggregated economic indicators from national statistical agencies. No animals, human participants, plants, microbe strains, cell lines, or primary cell cultures were used. Therefore, information regarding species/strain, genotype, age/developmental stage, sex, maintenance/care, and institutional oversight is not applicable.

As no human participants were involved, issues related to sex, gender, ancestry, race, ethnicity, sample size allocation, or experimental group assignment do not apply. The study's results are based on aggregate environmental and economic data, and thus the influence of sex or gender on the findings is not relevant.

### Method details

#### Satellite NO_2_ data

NO_2_ VCDs (January 2019–December 2022) were derived from TROPOMI/Sentinel-5P Level 2 products.^23^ The retrieval employs Differential Optical Absorption Spectroscopy (DOAS), which quantifies NO_2_ by analyzing backscattered solar radiation in the 405–465 nm spectral window, isolating absorption features through wavelength-specific optical depth fitting.^24^ Tropospheric VCDs were then calculated using Air Mass Factors (AMFs) generated by the TM5-MP chemical transport model at 0.25° resolution, incorporating cloud fraction, aerosol optical depth, and terrain height to account for light path modulation—reducing retrieval uncertainties below 15% over urban targets.^25^^,^^26^

Data processing followed a rigorous protocol: Quality-controlled observations required Quality Assurance (QA) values >75% and solar zenith angles <70° to minimize retrieval errors. Valid daily VCDs were aggregated into monthly mean composites at 0.1° grid resolution, mitigating short-term meteorological noise. To isolate anthropogenic emissions, we excluded natural NO_2_ sources by filtering out grids with active wildfires detected by VIIRS thermal anomaly data (375 m resolution). This processing chain leverages TROPOMI’s synergy of DOAS precision and physics-based AMFs, enabling robust tracking of urban-industrial emission dynamics at sub-city scales.

To analyze the NO_2_ trends at the national scale, we computed the monthly national average concentration from the quality-filtered gridded data. Specifically, for each month, only grids with at least 5 valid observation days and a QA value greater than 75% were retained. The monthly national mean NO_2_ VCD was derived using an area-weighted averaging method. We chose to use the directly observed VCD rather than estimated total emissions for trend analysis because: (1) VCD is a direct indicator of environmental exposure; (2) high-resolution, real-time emission inventories are generally unavailable at the monthly or finer temporal scales required to match satellite observations; and (3) emission inventories lack the spatial heterogeneity information provided by satellite data.^27^^,^^28^ The area-weighted VCD provides a robust national metric whose temporal dynamics are driven primarily by anthropogenic emissions from dominant source regions. Furthermore, the two key post-processing steps we applied—temporal aggregation to monthly means and the subsequent removal of the dominant seasonal cycle via decomposition (Section quantification and statistical analysis)—are designed to mitigate the influence of short-term synoptic variability and isolate the longer-term emission-driven trend from meteorological noise.

#### Transport activity metrics

China’s Ministry of Transport provides authoritative monthly records of freight and passenger flows across all transport modalities (2019–2022). This includes highway freight ton-kilometers, highway passenger-kilometers, railway traffic, civil aviation volumes, and waterway cargo throughput—forming the most comprehensive public dataset for national-scale mobility analysis. Core metrics follow internationally standardized definitions: Freight intensity as ton-kilometers (cargo weight × distance traveled), passenger mobility as passenger-kilometers (passenger count × journey distance). These directly interface with China’s transport energy consumption statistical framework, enabling robust conversion of transport output into energy-based emission factors.

The dataset’s uninterrupted monthly granularity captures full COVID-19 disruption-recovery cycles, with lockdown anomalies verified against provincial policy announcements.^29^^,^^30^ This temporal continuity provides unique advantages for analyzing short-term emission responses to economic shocks—surpassing the limitations of lower-frequency economic indicators.

#### Industrial economic output data

Quarterly secondary industry GDP data (2019–2022) were sourced from the National Bureau of Statistics of China, adhering to the Three Industries Classification Standard.^31^^,^^32^ This sector comprises stationary NO_2_ sources: manufacturing, mining, construction, and utilities.^33^ Data underwent price index deflation to constant values—calculated as current-price value-added divided by sectoral price indices—eliminating inflation bias for longitudinal comparison.

Temporal refinement employed cubic spline interpolation to derive monthly estimates. This technique preserves the acceleration signatures of the quarterly trend by enforcing continuity of first and second derivatives at quarterly nodes, outperforming linear interpolation during volatile periods. It is important to note that this interpolation creates a mathematically smooth series; it does not replicate high-frequency, within-quarter fluctuations present in raw economic activity, such as the pronounced production decline during the annual Spring Festival period. This is a necessary limitation given the absence of officially published, consistent monthly GDP data at the national level. We posit that for analyzing pollutant responses to economic trends over monthly to quarterly timescales, the overarching trend captured by this method remains informative.

### Quantification and statistical analysis

We integrate monthly data aggregation, STL, Granger causality, and CCF into a cascaded analytical framework. First, daily observations are aggregated to monthly means to reduce high-frequency synoptic noise. STL is then applied to decompose the monthly NO_2_ series into seasonal, residual and trend components.^34^ The seasonal component captures recurrent patterns strongly influenced by meteorological conditions (e.g. boundary layer height, temperature) and periodic anthropogenic activities (e.g. winter heating). Its removal reduces the dominant influence of seasonal meteorology. The residual component contains higher-frequency variability, including sub-seasonal meteorological noise and other unexplained short-term fluctuations. The long-term trend component—purified of both seasonal cycles and residual noise—serves as the primary signal for subsequent Granger causality and correlation analyses with economic indicators.

Granger causality with BIC-optimized lags quantifies sectoral contributions while modeling transport-industrial synergies via cross-lagged terms.^35^ CCF analysis maps policy-to-emission lags using small-sample-adjusted significance thresholds. This sequence solves the core challenge of isolating policy-relevant signals in dynamic economies.^36^

China-specific customizations ensure validity: Regionally parameterized STL windows (90-day north/60-day south) address heating policy heterogeneity. Granger models incorporate freight-industry interaction terms to decode synergistic effects. CCF confidence intervals are Monte Carlo-calibrated for 48-month environmental series—critical for robust lag detection in rapid transition contexts.

This integrated framework is designed to systematically uncover and quantify the temporal associations and predictive relationships between sectoral activities and NO_2_ concentrations. Specifically, our correlation-based analysis of detrended series aims to diagnose high-frequency, short-term associative dynamics and emission intensity signals between activity and pollution. This provides a complementary perspective to formal, long-term decoupling indices (e.g. the Tapio index), which focus on the elasticity between growth rates of output and emissions. Our approach captures high-frequency co-variation and intensity changes, thereby identifying priority sectors and dynamic patterns for immediate policy scrutiny. The framework’s policy assessment interfaces should be interpreted as revealing suggestive, correlational evidence: command-control interventions may be inferred from trend-slope changes; market incentives monitored via residual impulse responses; structural reforms suggested by shifts in predictive linkage strength. It is important to note that while Granger causality indicates predictive precedence and CCF reveals lead-lag correlations, neither method establishes structural or counterfactual causality to definitively isolate policy impacts from concurrent confounders. Nonetheless, this architecture formalizes a evidence-based surveillance paradigm—translating China’s policy experimentation experience into a transferable tool for developing nations to identify key linkages and generate hypotheses for subsequent causal evaluation.

## Supplemental information

Document S1. Original code for STL decomposition, Granger causality, and cross-correlation function analysis, related to STAR Methods This document contains the complete set of Python scripts used to process satellite NO_2_ data, interpolate economic indicators, perform STL, conduct Granger causality tests with BIC-optimized lags, and compute CCFs.
