# Supplementary material for: Divergent decoupling pathways for economic growth and NO2 emissions in China revealed by satellite observations
Source: iScience. 2026 Mar 12;29(4):115352. doi: 10.1016/j.isci.2026.115352 (PMC13053765; doi:10.1016/j.isci.2026.115352)
Supplement: Document S1. Original code for STL decomposition, Granger causality, and cross-correlation function analysis, related to STAR Methods — This document contains the complete set of Python scripts used to process satellite NO2 data, interpolate economic indicators, perform STL, conduct Granger causality tests with BIC-optimized lags, and compute CCFs. [file mmc1.pdf]

iScience, Volume *29*

Supplemental information

Divergent decoupling pathways for economic growth and NO_2 emissions in China revealed by satellite observations

Jianbin Gu, Ziwei Chai, Ruimin Deng, Ying Zhang, Mingge Li, and Liangfu Chen

Lead Contact: Ziwei Chai, Email: Chinaziweich@163.com

Document S1. Original code for STL decomposition, Granger causality, and cross-correlation function analysis, related to STAR Methods.

```
#!/usr/bin/env python3
# -*- coding: utf-8 -*-
"""
Supplementary Code for:
"Divergent Decoupling Pathways for Economic Growth and NO2 Emissions in China
  Revealed by Satellite Observations"

This script contains the essential analysis functions:
- Process TROPOMI NO2 satellite data to monthly national averages
- Interpolate quarterly industrial GDP to monthly frequency
- Seasonal-Trend decomposition (STL) of NO2 time series
- Granger causality tests with BIC-optimized lag selection
- Cross-correlation function (CCF) analysis

Dependencies:
    numpy, pandas, scipy, statsmodels, xarray, netCDF4 (optional for netCDF)
"""


import numpy as np
import pandas as pd
from scipy.interpolate import CubicSpline
from statsmodels.tsa.seasonal import STL
from statsmodels.tsa.stattools import grangercausalitytests, ccf
import warnings
warnings.filterwarnings("ignore")
```

```
# ------------------------------
# 1. Satellite NO2 Data Processing
# ------------------------------

def load_no2_data(tropomi_file_pattern, start_date='2019-01', end_date='2022-12'):
    """
    Load TROPOMI NO2 tropospheric vertical column densities and compute
    monthly national averages.

    This is a template function. In practice, TROPOMI Level 2 data are in netCDF
    format. The function demonstrates the workflow:
        - Read multiple files
        - Apply quality filtering (QA > 75%, solar zenith angle < 70°)
        - Aggregate daily data to monthly 0.1°  grids
        - Exclude wildfire-affected grids using VIIRS data (not shown)
        - Calculate area-weighted monthly mean over China

    Parameters
    ----------
    tropomi_file_pattern : str
        Glob pattern for TROPOMI netCDF files.
    start_date, end_date : str
        Period of interest.

    Returns
    -------
    pd.Series
        Monthly national mean NO2 VCD (index=period, values=10^15 molec/cm²)
    """
```

```
    # --- Placeholder for actual file reading ---
    # In real code, use xarray.open_mfdataset or loop over netCDF files.
    # Example:
    # import xarray as xr
    # ds = xr.open_mfdataset(tropomi_file_pattern, combine='by_coords')
    # Apply QA filter: ds = ds.where(ds.qa_value > 0.75)
    # Filter SZA: ds = ds.where(ds.solar_zenith_angle < 70)
    # Aggregate to 0.1° grid (resample), then to monthly means.
    # Weight by grid cell area (cos(latitude)).
    # Mask wildfire pixels using VIIRS (not implemented here).
    # ---

    # For demonstration, generate synthetic monthly NO2 data (2019-2022)
    dates = pd.date_range(start=start_date, end=end_date, freq='MS')
    np.random.seed(42)
    trend = np.linspace(1.2, 1.0, len(dates))  # slight downward trend
    seasonal = 0.3 * np.sin(2 * np.pi * (dates.month / 12) - 1.5)
    noise = np.random.normal(0, 0.05, len(dates))
    no2_values = trend + seasonal + noise + 0.8
    no2_series = pd.Series(no2_values, index=dates, name='NO2_VCD')
    print("Synthetic NO2 data generated. Replace with actual data reader.")
    return no2_series


# ------------------------------------
# 2. Economic Indicators Interpolation
# ------------------------------------

def interpolate_quarterly_gdp(quarterly_gdp_csv, start_month='2019-01', end_month='2022-12'):
    """
```

```
Interpolate quarterly secondary industry GDP to monthly frequency
using cubic spline interpolation.

Parameters
----------
quarterly_gdp_csv : str
    Path to CSV file with columns 'date' (quarter end, e.g., 2019-03-31)
    and 'value' (billion CNY, constant prices).
start_month, end_month : str
    Period for monthly interpolation.

Returns
-------
pd.Series
    Monthly GDP values, index=month start.
"""
# Read quarterly data
df_q = pd.read_csv(quarterly_gdp_csv, parse_dates=['date'])
df_q = df_q.sort_values('date')
# Convert to numeric timestamps for spline
x = df_q['date'].map(pd.Timestamp.toordinal).values
y = df_q['value'].values

# Create monthly dates
monthly_dates = pd.date_range(start=start_month, end=end_month, freq='MS')
x_monthly = monthly_dates.map(pd.Timestamp.toordinal).values

# Cubic spline interpolation
cs = CubicSpline(x, y, bc_type='natural')
y_monthly = cs(x_monthly)
```

```python
    # Ensure non-negative
    y_monthly = np.maximum(y_monthly, 0)

    return pd.Series(y_monthly, index=monthly_dates, name='GDP_monthly')

def load_transport_data(transport_csv, variable='freight_tkm'):
    """
    Load monthly transport data from Ministry of Transport.
    Expected CSV columns: 'date' (YYYY-MM-DD or YYYY-MM), variable column.
    """
    df = pd.read_csv(transport_csv, parse_dates=['date'])
    df = df.set_index('date').sort_index()
    # Ensure monthly frequency (month start)
    monthly = df[variable].resample('MS').mean()
    return monthly


# -------------------------------------------
# 3. Seasonal-Trend Decomposition (STL)
# -------------------------------------------

def stl_decompose(series, period=12, robust=True):
    """
    Perform STL decomposition on a monthly time series.

    Parameters
    ----------
    series : pd.Series
        Monthly data with datetime index.
```

```
    period : int
        Seasonal period (12 for monthly).
    robust : bool
        If True, use robust fitting.

    Returns
    -------
    pd.DataFrame
        Columns: 'observed', 'trend', 'seasonal', 'resid'.
    """
    stl = STL(series, period=period, robust=robust)
    res = stl.fit()
    df = pd.DataFrame({
        'observed': series,
        'trend': res.trend,
        'seasonal': res.seasonal,
        'resid': res.resid
    }, index=series.index)
    return df


# --------------------------------------------
# 4. Granger Causality with BIC-optimal lags
# --------------------------------------------

def granger_causality_bic(y, x, maxlag=12):
    """
    Test whether time series x Granger-causes y, with automatic lag selection
    based on Bayesian Information Criterion (BIC).
```

```
Parameters
----------
y, x : pd.Series
    Two time series (must be aligned, stationary).
maxlag : int
    Maximum number of lags to consider.

Returns
-------
dict
    Contains:
    - 'best_lag': lag with minimum BIC
    - 'bic_values': BIC for each lag
    - 'test_results': Granger test result at best_lag
    - 'p_value': p-value at best_lag
"""
# Ensure same index, drop NaN
data = pd.concat([y, x], axis=1).dropna()
if len(data) < maxlag + 1:
    raise ValueError("Insufficient observations after dropping NaNs.")

# Granger causality tests over lags 1..maxlag
test_res = grangercausalitytests(data[[x.name, y.name]], maxlag=maxlag, verbose=False)

bic_values = {}
for lag in range(1, maxlag+1):
    # Extract BIC from the test result for 'ssr_ftest' (lag is key)
    # The structure: test_res[lag][0]['ssr_ftest'] gives (F, p, df1, df2)
    # We need BIC from the unrestricted model; stored in test_res[lag][0]['lrtest']?
```

```
        # Actually, grangercausalitytests returns a dict with 'ssr_ftest', 'ssr_chi2test', 'lrtest', 'params_ftest'
        # The BIC for the unrestricted model is in test_res[lag][0]['ssr_ftest']? No.
        # Simpler: Re-fit OLS and compute BIC manually:
        from statsmodels.tsa.tsatools import lagmat
        from statsmodels.regression.linear_model import OLS

        # Prepare lags of x and y
        x_lags = lagmat(x, lag)
        y_lags = lagmat(y, lag)
        # Unrestricted model: predict y using lags of y and x
        X_unres = np.column_stack([np.ones(len(x_lags)), x_lags, y_lags])
        y_aligned = y.iloc[lag:]
        # Handle alignment (lagmat prepends NaN)
        valid = ~np.isnan(X_unres).any(axis=1)
        X_unres = X_unres[valid]
        y_aligned = y_aligned[valid]

        model_unres = OLS(y_aligned, X_unres).fit()
        bic_values[lag] = model_unres.bic

    # Find lag with minimum BIC
    best_lag = min(bic_values, key=bic_values.get)
    # Run test again at best_lag to get p-value
    best_test = grangercausalitytests(data[[x.name, y.name]], maxlag=best_lag, verbose=False)
    p_value = best_test[best_lag][0]['ssr_ftest'][1]    # p-value from F-test

    return {
        'best_lag': best_lag,
        'bic_values': bic_values,
```

```
            'test_results': best_test[best_lag],
            'p_value': p_value
        }


# --------------------------------------------
# 5. Cross-Correlation Function (CCF)
# --------------------------------------------

def cross_correlation_analysis(x, y, max_lag=25):
    """
    Compute cross-correlation function between two time series.

    Parameters
    ----------
    x, y : pd.Series
        Time series (must be aligned, should be detrended for meaningful results).
    max_lag : int
        Maximum lag (positive and negative).

    Returns
    -------
    pd.DataFrame
        Index = lags from -max_lag to +max_lag, columns = ['corr', 'ci_lower', 'ci_upper'].
        Confidence intervals (95%) based on Bartlett's formula.
    """
    # Align and drop NaN
    data = pd.concat([x, y], axis=1).dropna()
    x_vals = data.iloc[:, 0].values
    y_vals = data.iloc[:, 1].values
```

```
# Compute CCF (statsmodels ccf returns correlations for lags 0..max_lag)
# We need both positive and negative lags.
# ccf(x, y) gives corr(x_{t}, y_{t+k}) for k=0..nlags.
# To get negative lags, swap x and y.
nlags = max_lag
ccf_pos = ccf(x_vals, y_vals, adjusted=False)    # length nlags+1
ccf_neg = ccf(y_vals, x_vals, adjusted=False)    # gives correlations for negative lags

# Construct full array from -max_lag to +max_lag
lags = np.arange(-max_lag, max_lag + 1)
corr = np.zeros(len(lags))
# positive lags (including 0)
pos_len = min(max_lag, len(ccf_pos)-1)
corr[max_lag : max_lag + pos_len + 1] = ccf_pos[:pos_len+1]
# negative lags
neg_len = min(max_lag, len(ccf_neg)-1)
# ccf_neg[0] is lag 0, we need lag -1 = ccf_neg[1], etc.
corr[max_lag - neg_len : max_lag] = ccf_neg[1:neg_len+1][::-1]

# Approximate 95% confidence interval:  ±  1.96 / sqrt(n)
n = len(x_vals)
ci = 1.96 / np.sqrt(n)

df_ccf = pd.DataFrame({
    'corr': corr,
    'ci_lower': corr - ci,
    'ci_upper': corr + ci
}, index=lags)
df_ccf.index.name = 'lag'
```

```
    return df_ccf


# ------------------------------------------
# Example Workflow (Main)
# ------------------------------------------

def main():
    """Demonstrate the full analysis pipeline using synthetic data."""
    print("=== Supplementary Code: NO2 Decoupling Analysis ===")

    # -------------------------
    # Step 1: Load and preprocess NO2 data
    # -------------------------
    # In practice, replace with actual TROPOMI file pattern
    no2_raw = load_no2_data('dummy_path')
    print(f"\nNO2 data (monthly mean): {no2_raw.index[0]} to {no2_raw.index[-1]}")

    # -------------------------
    # Step 2: Load economic data
    # -------------------------
    # For demonstration, create synthetic transport and GDP data.
    # In practice, read from official CSV files.
    dates = pd.date_range('2019-01', '2022-12', freq='MS')
    np.random.seed(123)

    # Freight transport (ton-km)
    freight_trend = np.linspace(200, 220, len(dates))
    freight_seasonal = 15 * np.sin(2 * np.pi * (dates.month / 12))
    freight_noise = np.random.normal(0, 5, len(dates))
```

```
freight = freight_trend + freight_seasonal + freight_noise
freight_series = pd.Series(freight, index=dates, name='freight_tkm')

# Passenger transport (passenger-km)
passenger_trend = np.linspace(100, 80, len(dates))    # decline due to COVID
passenger_seasonal = 10 * np.sin(2 * np.pi * (dates.month / 12))
passenger_noise = np.random.normal(0, 4, len(dates))
passenger = passenger_trend + passenger_seasonal + passenger_noise
passenger_series = pd.Series(passenger, index=dates, name='passenger_pkm')

# Industrial GDP (quarterly -> monthly)
# Create synthetic quarterly data
q_dates = pd.date_range('2019-03-31', '2022-12-31', freq='Q')
gdp_quarterly = pd.Series(
    [2100, 2150, 2200, 2000, 2100, 2250, 2300, 2350, 2400, 2450, 2500, 2550],
    index=q_dates, name='GDP'
)
gdp_monthly = interpolate_quarterly_gdp(quarterly_gdp_csv=None)    # placeholder
# Override with synthetic interpolation
gdp_monthly = pd.Series(
    np.linspace(2100, 2550, len(dates)),
    index=dates, name='GDP_monthly'
)
print("\nEconomic indicators loaded (synthetic).")

# --------------------------
# Step 3: STL decomposition of NO2
# --------------------------
no2_stl = stl_decompose(no2_raw)
no2_trend = no2_stl['trend']
```

```
print("\nSTL decomposition completed. Trend component extracted.")

# -------------------------
# Step 4: Granger causality tests
# -------------------------
# Use detrended NO2 trend? The paper uses the trend component.
# But for Granger test, both series should be stationary.
# Here we use the NO2 trend (non-stationary) as in the paper?
# Actually the paper says "detrended economic activities and the NO2 trend component".
# We'll assume the NO2 trend is used as is (or differenced to achieve stationarity).
# For simplicity, we compute on the trend series (may need differencing).
# We'll follow the paper: use the STL trend component.

print("\nPerforming Granger causality tests (BIC-optimized lags)...")

# Freight -> NO2
res_freight = granger_causality_bic(no2_trend, freight_series, maxlag=6)
print(f"Freight -> NO2: best lag={res_freight['best_lag']}, p={res_freight['p_value']:.4f}")

# Passenger -> NO2
res_passenger = granger_causality_bic(no2_trend, passenger_series, maxlag=6)
print(f"Passenger -> NO2: best lag={res_passenger['best_lag']}, p={res_passenger['p_value']:.4f}")

# Industry GDP -> NO2
res_ind = granger_causality_bic(no2_trend, gdp_monthly, maxlag=6)
print(f"Industry GDP -> NO2: best lag={res_ind['best_lag']}, p={res_ind['p_value']:.4f}")

# -------------------------
# Step 5: Cross-correlation functions
```

```
    # --------------------------
    print("\nComputing cross-correlation functions (CCF)...")
    # Use detrended series (remove long-term trend to focus on short-term covariation)
    # For NO2, we can use the residual component after STL.
    no2_detrended = no2_stl['resid'] + no2_stl['seasonal']    # or just resid?
    # Actually to remove trend, use resid+seasonal or just resid?
    # For economic indicators, also detrend by differencing or linear detrend.
    # Here we simply use the original series (synthetic have trend)  –  in practice pre-whiten.

    # For demonstration, we use the raw series (not detrended).
    # In actual analysis, you should remove deterministic trends first.

    # Freight vs NO2
    ccf_freight = cross_correlation_analysis(freight_series, no2_raw, max_lag=12)
    print("CCF freight-NO2 computed.")

    # Passenger vs NO2
    ccf_passenger = cross_correlation_analysis(passenger_series, no2_raw, max_lag=12)
    print("CCF passenger-NO2 computed.")

    # Industry GDP vs NO2
    ccf_industry = cross_correlation_analysis(gdp_monthly, no2_raw, max_lag=12)
    print("CCF industry-NO2 computed.")

    print("\n=== End of demonstration. ===")


if __name__ == '__main__':
    main()
```
